# Myeloproliferative neoplasm with eosinophilia and T-lymphoblastic lymphoma with ETV6–LYN gene fusion

**DOI:** 10.1038/bcj.2016.11

**Published:** 2016-04-08

**Authors:** N Telford, S Alexander, O J McGinn, M Williams, K M Wood, A Bloor, V Saha

**Affiliations:** 1 Oncology Cytogenetics, The Christie Pathology Partnership, The Christie NHS Foundation Trust, Manchester, UK; 2 Children's Cancer Group, Centre for Paediatric, Teenage and Young Adult Cancer, Institute of Cancer, University of Manchester, Manchester, UK; 3 Leukaemia Biology Group, Institute of Cancer, University of Manchester, Manchester, UK; 4 Department of Cellular Pathology, Royal Victoria Infirmary, The Newcastle upon Tyne Hospitals NHS Foundation Trust, Newcastle Upon Tyne, UK; 5 Haematology and Transplant Unit, The Christie NHS Foundation Trust, Manchester, UK; 6 Tata Translational Cancer Research Centre, Tata Medical Center, Kolkata, India

Myeloproliferative neoplasms (MPNs) are a heterogeneous group of clonal haematopoietic stem cell disorders characterised by proliferation and maturation of different myeloid cell lineages. Classical MPNs are largely characterised by somatic mutation of JAK2, CALR or occasionally MPL genes.^1, 2^ MPNs can develop from chromosomal translocations forming gene fusions, involving constitutive activation and aberrant expression of tyrosine kinase (TK). Other than the BCR-ABL1 rearrangement in chronic myeloid leukaemia, gene fusions involving TK proto-oncogenes are rare in MPN but when detected, provide compelling disease-specific therapeutic targets.^3^ Myeloproliferative and lymphoid neoplasms with eosinophilia and specific TK gene fusions are a recently described, rare entity of clinical significance due to varying responsiveness to tyrosine kinase inhibitors (TKIs).^4^ The three cytogenetic categories identified, involving fusions of PDGFRA, PDGFRB or FGFR1 with different partner genes, are thought to affect a pluripotent stem cell and have variable presentation as MPN with eosinophilia, occasionally with a T-lymphoid disease component. Excellent response rates and long-term clinical outcomes have been reported with abnormalities of PDGFRA and PDGFRB treated with imatinib, whereas disorders with FGFR1 and JAK2 gene fusions are resistant to imatinib and other TKIs. We present a case of a disease fitting within this clinical and haematological spectrum, with a chromosomal rearrangement between chromosomes 8 and 12, bearing ETV6–LYN gene fusion.

A 46-year-old man, of Bangladeshi origin, presented with a 3-month history of progressive swelling in his groin from bilateral inguinal lymphadenopathy, associated with lethargy and sweats. Histology from an excision biopsy of a left-sided inguinal node diagnosed a precursor T-lymphoblastic lymphoma (T-LBL; CD2+, CD3+, CD5+, CD10−, CD20+, focal weak CD79a, strong nuclear Tdt +ve, cyclin D1−, bcl2 weak, bcl6−, Ki67 80% proliferative fraction). Peripheral blood showed a leuco-erythroblastic picture with high white blood cell count (17.2 × 10^9^/l) with eosinophilia. Bone marrow (BM) aspirate and trephine biopsies showed prominent myeloid hyperplasia with eosinophilia, no excess of blasts but with a 10% interstitial infiltrate of T-cell lymphoma. The patient received dexamethasone, daunorubicin, vincristine and asparaginase (UKALLXII trial regime), which eradicated the lymphoma, but failed to control the myeloproliferative component. Reinduction with FLA–IDA (fludarabine, high-dose cytarabine and idarubicin) reduced the WCC, but did not achieve complete cytogenetic remission (see below). After approximately 5 months, the disease progressed to refractory acute myeloid leukaemia (AML) and the patient died 7 months from first presentation.

G-banded cytogenetic analysis of the diagnostic BM aspirate showed an apparent translocation between the long arm of chromosome 8 and short arm of chromosome 12, although the resolution was not sufficient to confidently confirm the breakpoint on chromosome 8 (Figure 1a). The majority of cells also showed one or two additional copies of the derivative chromosome 8. Fluorescence *in situ* hybridisation (FISH) using ETV6 gene probes (Vysis, Downer's Grove, IL, USA) showed ETV6 gene rearrangement in 88/100 fixed cells from the BM aspirate and from a paraffin-embedded tissue section from the lymph node infiltrated with T-LBL, indicating a clonal relationship between the two tissues of different morphology (Figures 1b and c). FISH with ETV6 gene probes showed a normal signal pattern in cells from a buccal smear and was also normal on BM cells using BCR-ABL1, KMT2A, PDGFRA, PDGFRB and FGFR1 probes (Vysis). Reverse transcriptase PCR showed no evidence of ETV6–NCOA2 gene rearrangement, initially thought to be a candidate ETV6 fusion partner.^5^ DNA from presentation BM and a sample during morphological remission were hybridised to Affymetrix Genome-Wide Human SNP Array 6.0 (Affymetrix, Santa Clara, CA, USA), and copy number variation was compared (Supplementary Figure 3a). Owing to the chromosome imbalance from the additional copies of the abnormal chromosomes 8, the segments involved in the translocation were demonstrated as DNA copy number change and the breakpoints were defined in detail. Despite the presence of a recognisable breakpoint within ETV6 on chromosome 12, the most distal break on chromosome 8 was not within the open reading frame of a recognised gene (Supplementary Figure 3b). Sites of deletion and amplification of DNA sequences suggested greater molecular disruption of the region and possibly implicated localised chromothrypsis on chromosome 8. The LYN TK gene was found to be at the border of a deletion of ~46 kb at 8q12.1 (Supplementary Figure 4). An ETV6–LYN gene fusion was confirmed by RT-PCR using primers as previously published^6^ (Supplementary Method 1 and Figure 5) and capillary sequencing of the PCR product defined the molecular junction (Supplementary Figure 6). This suggested a complex chromosomal rearrangement comprising of at least three breaks. In its simplest possible form, a 22.13 Mb inversion was implicated (between positions 56 864 550 and 78 992 979 bp human genome assembly hg19 on chromosome 8, flanked by deletions of ~46 kb at 8q12.1 and ~167kb at 8q21.1). This brings LYN and ETV6 into juxtaposition in the correct 5′–3′ orientation to create the in-frame chimaeric fusion gene on the derived chromosome 8 and is necessary owing to the opposite orientation of the genes to their centromeres (Supplementary Figure 7).

The transforming potential of ETV6–LYN gene fusion in haematological malignancy has been demonstrated previously from a study screening artificially constructed ETV6 fusion partners from complementary DNA.^7^ However, there is only one previous clinical case report of ETV6–LYN fusion gene, with myelofibrosis, resulting from an interchromosomal insertion, ins(12;8)(p13;q11q21), again demonstrating that a three-break rearrangement is required for the fusion to form.^8^ The genomic breakpoints in the current case yield a chimaeric mRNA with fusion of exons 5 in ETV6 to exon 8 in LYN, identical to the previous report. As multiple breaks are required to create the reported fusion, it may explain why the rearrangement appears rare, but also suggests that some chromosomal rearrangements may be cryptic and that ETV6–LYN may be underreported. One further case of eosinophilic MPN/T-LBL with t(8;12), but without confirmation of ETV6–LYN, shows remarkably similar clinical features to the current case.^9^ All three patients were young adult males, with a myeloproliferative disease in the BM, two having peripheral T-LBL. All three patients were refractory to intensive chemotherapy or stem cell transplant and showed a rapid progression to terminal AML (Supplementary Table 1). Patients with the fusion appear to be desperate candidates for novel approaches to treatment in attempt to improve outcomes.

ETV6 (ets variant 6, formerly TEL) encodes a ubiquitously expressed nuclear protein, acting as a signal-dependent transcription repressor, mediating cell proliferation and differentiation, and is necessary for the maintenance of haematopoiesis of all cell lineages. ETV6 is recognised as having an important role in leukaemogenesis through different oncogenic mechanisms, including the formation of gene fusions with up to 30 partner genes.^10^ ETV6 partners with non-receptor TK genes, in which fusion typically occurs at exon 4 or 5 of ETV6, with retention of the activating helix-loop-helix oligodimersation (pointed (PNT)) domain and the TK functionality of its partner.^11^ LYN (v-yes-1 Yamaguchi sarcoma viral related oncogene homologue, formerly JTK8) is one of nine members of the Src family of membrane-associated, non-receptor TKs, which fulfils a critical role in the proliferative response of neoplastic myeloid cells to cytokine signalling. LYN contains kinase (SH1), Src homology (SH2 and SH3) and unique (SH4) domains.^12^ The ETV6–LYN gene fusion is typical of the non-receptor TK translocations in that the PNT domain of ETV6 activates LYN by protein oligomerization and autophosphorylation of the kinase, stimulating STAT3 and STAT5, resulting in neoplastic transformation to myeloproliferative disease, independent of upstream JAK2 activation.^6^

Lyn is consistently expressed in AML^13^ and in imatinib-resistant chronic myeloid leukaemia,^14^ and is therefore a compelling pharmacologic target for leukaemia therapy. Inhibition of Lyn kinase activity has been demonstrated using Src kinase inhibitors, resulting in control of STAT5 activation with induction of apoptosis and suppression of leukaemic cell growth. The presence of ETV6–LYN may offer a valid biomarker predicting sensitivity of the disease to TKI. The single previous case of ETV6–LYN was insensitive to imatinib clinically^8^ and experimentally sensitive to dasatinib.^6, 8^ We investigated the *in vitro* sensitivity to dasatinib, imatinib, bosutinib and nilotinib in a series of experiments on fresh-frozen, stored cells from the patient's BM at diagnosis and in morphological remission as control (Methods, see Supplementary Material 5). The patient's diagnostic marrow cells appeared to have erythroid burst-forming units growing independently of cytokines, in keeping with an MPN. The primary cells from the diagnostic marrow showed increased sensitivity to dasatinib compared with the remission marrow (Figure 2a), which is consistent with previous observations. A response to imatinib was also apparent, whilst there was no observed benefit of using nilotinib and bosutinib (Figures 2a and b). Sensitivity of cells from the diagnostic BM to various concentrations of dasatinib (0.1, 1 and 10 μM) showed that there was a significant effect at all doses compared with remission and untreated cells, but that there was no significant difference between different concentrations (Figure 2b and Supplementary Information; Figure 8) and so no dose–response relationship was apparent. Dasatinib is a small molecule, spectrum TKI, known to inhibit the Src family kinases, including Lyn, as well as Abl1 and receptor TKs, and this result was expected. The superiority of dasatinib over bosutinib, a known Src inhibitor, is unclear but could result from the broader spectrum of dasatinib targeting co-expressed Src kinases having homology with LYN, leading to combined inhibition of other interconnected signalling pathways. Nilotinib and imatinib have similar selectivity profiles and do not inhibit Src kinases. The previous evidence of *in vitro* failure of imatinib therapy and repeated experimental evidence of cell sensitivity to dasatinib makes this TKI a more attractive candidate therapy for this disease, as a single agent or with chemotherapy to enhance sensitivity.^15^

In summary, MPN with eosinophilia and T-LBL, involving rearrangement of chromosomes 8 and 12 resulting in ETV6–LYN gene fusion, is a rare entity consistent with the World Health Organization category of myeloproliferative and lymphoid neoplasms with eosinophilia and specific gene fusions. On the basis of the three available cases, this represents a high-risk disease with fibrotic tendency, resistance to conventional therapy and rapid progression to AML. Such patients may benefit from dasatinib monotherapy or as an adjunctive therapy with conventional cytotoxics.

## Figures and Tables

**Figure 1:**
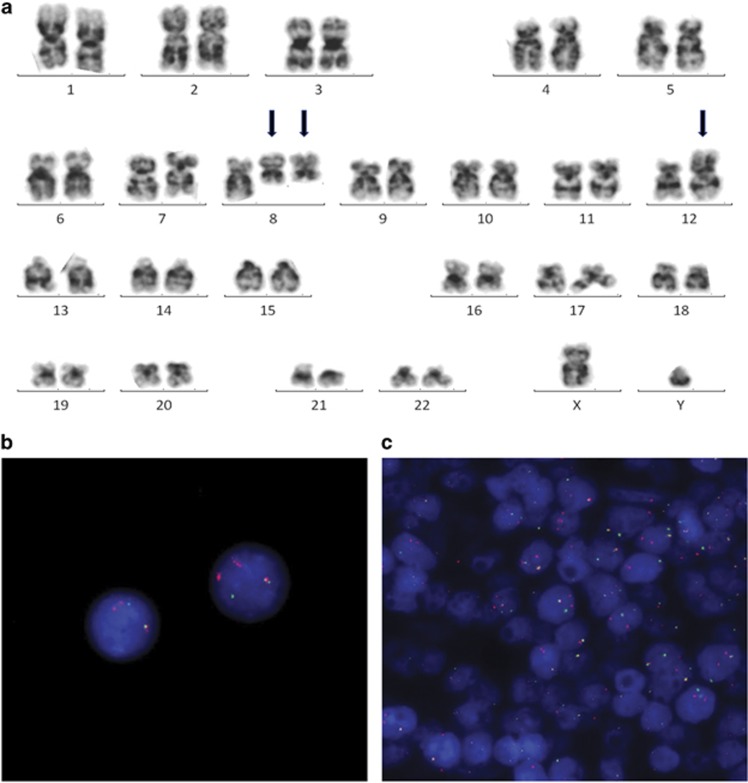
(**a**) G-banded karyogram from the diagnostic bone marrow showing the rearrangement between the long arm of chromosome 8 and short arm of chromosome 12 (arrowed), with an extra copy of the derivative chromosome 8, International System for Human Cytogenetic Nomenclature (ISCN) karyotype refined as: 46,XY,der(8)inv(q12.1q21.1)t(8;12)(q12.1;p13),der(12)t(8;12)(q12.1;p13)[2]/47,sl,+der(8)inv(8)t(8;12)[5]/48,sdl1,+der(8)inv(8)t(8;12)[2]/46,XY[2]. Fluorescence *in situ* hybridisation (FISH) with ETV6 break apart gene probes showing split red and green signals, and extra red signal from the duplicated der(8) in interphase cell nuclei from the bone marrow aspirate (**b**) and on the paraffin-embedded tissue section from the lymph node biopsy (**c**).

**Figure 2:**
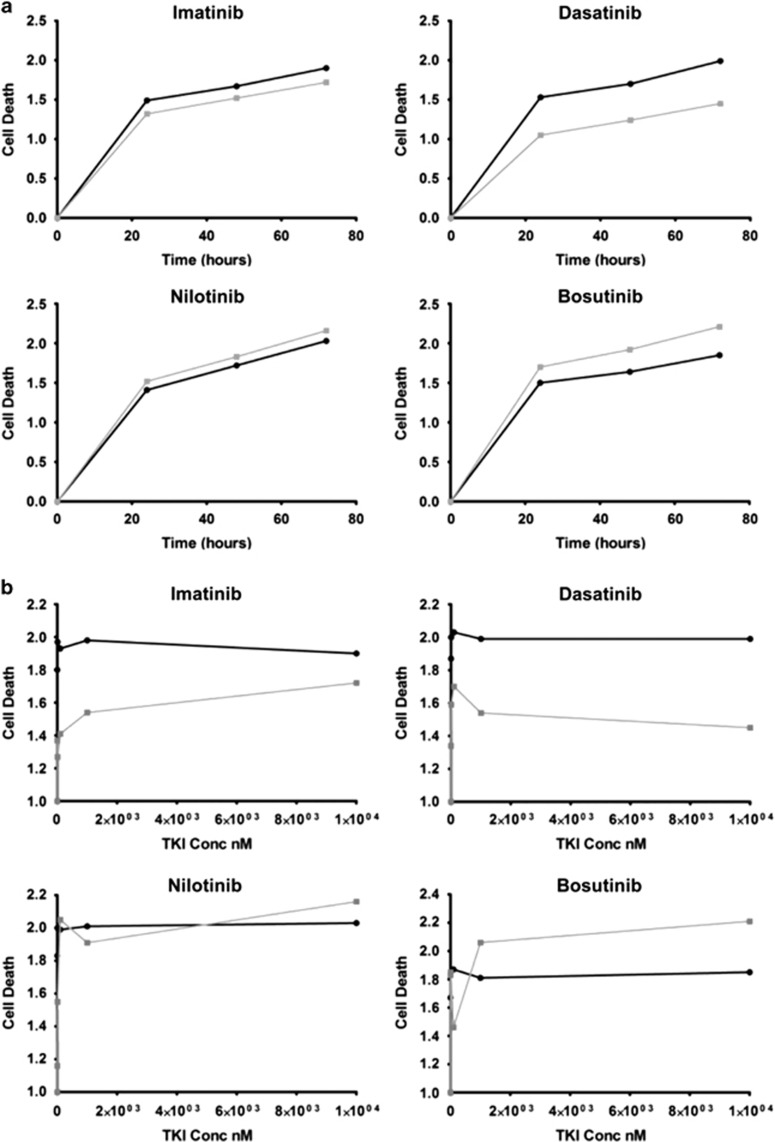
Charts showing relative cell death (data are arbitrary fluorescence units, double normalised by dividing by the maximum death unit and then expressed relative to the untreated), measured over 72 h at the maximum concentration of TKI (**a**) and at different concentrations of TKI (nM) (**b**). The patient's diagnostic BM cells (black curve) show varying sensitivity to the TKIs, imatinib, dasatinib, nilotinib and bosutinib compared with the remission marrow (grey curve).
