# Supplementary material for: Myeloproliferative neoplasm with eosinophilia and T-lymphoblastic lymphoma with ETV6–LYN gene fusion
Source: Blood Cancer J. 2016 Apr 8;6(4):e412–. doi: 10.1038/bcj.2016.11 (PMC4855251; doi:10.1038/bcj.2016.11)
Supplement: Supplementary Information [file bcj201611x1.pdf]

Myeloproliferative neoplasm with eosinophilia and T-lymphoblastic lymphoma with ETV6-LYN gene fusion shows in vitro sensitivity to the tyrosine kinase inhibitor dasatinib.

Supplementary Information

Figure 3a

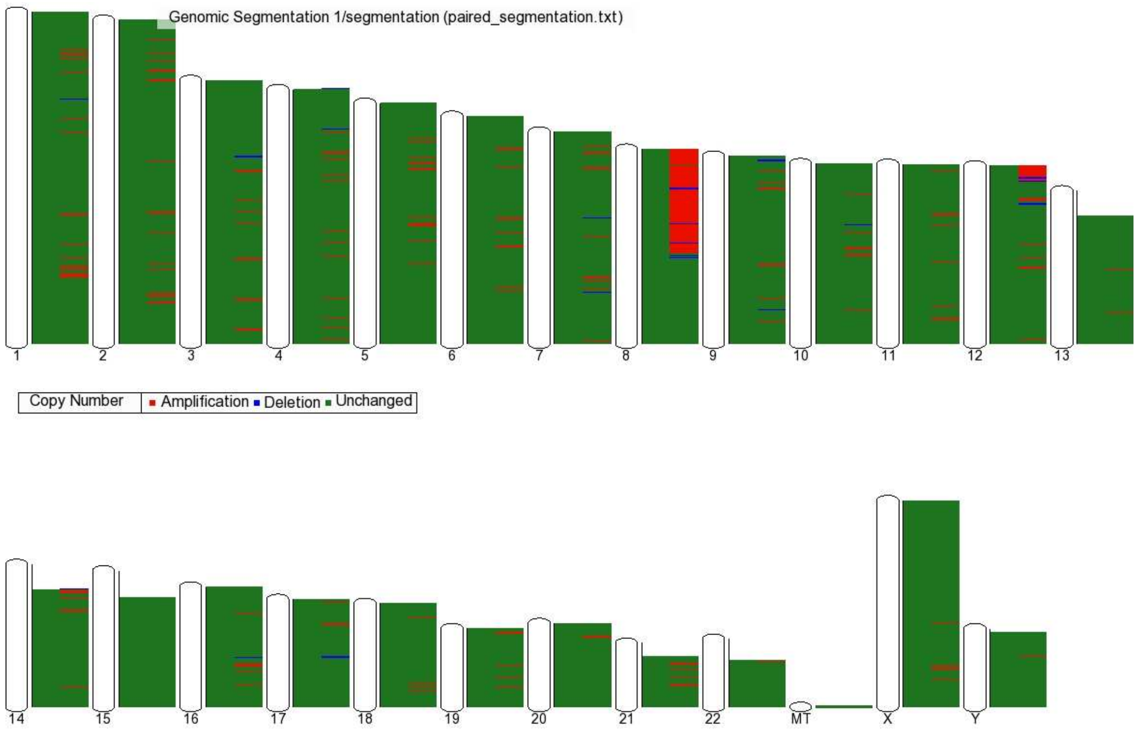

Figure 3b

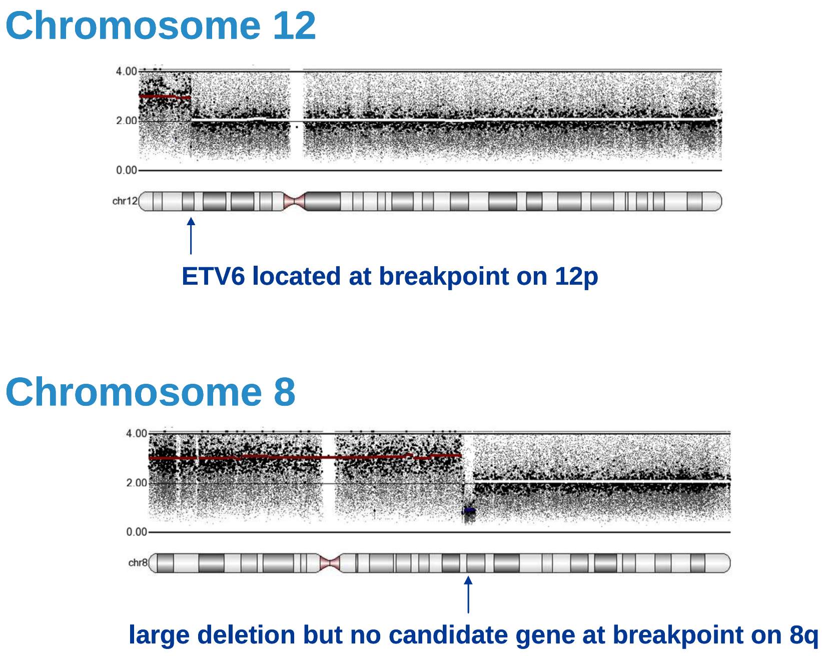

Figure 3a. Diagrammatic representation of genomic copy number variation of the diagnostic bone marrow (right hand side of the green bar for each chromosome) compared to sample in morphological remission. Amplification is shown in red and deletion in blue), showing extensive gain of chromosome 8 and 12 material from the extra copy of the der(8).

Figure 3b. Traces of SNP copy number along the length of chromosomes 8 and 12, showing demarcation of chromosomal breakpoints.

Figure 4. Plot of copy number of chromosome 8 sequences from Affymetrix Genome-Wide Human SNP Array 6.0 data. Chromosomal position (in Mbp from the p terminus) is displayed on the x axis and SNP copy number on the y axis. Three copies of chromosome 8 sequences (from pter to 8q21.12) are consistent with sequences on the extra der(8) whereas two copies (from 8q21.12 to 8qter) are consistent with regions present on the der(12) and unrearranged chromosome. This cannot predict the orientation of segments of chromosome 8. However, for LYN to be in the correct alignment with the break within ETV6 from chromosome 12, at least an inversion of 22.13 Mb (between 8q12.1 and 8q21.12) has occurred. Other genomic imbalances are apparent; peaks of 6 copies (implying microduplications on the additional der(8)) and troughs of single copies (implying deletion of chromosome 8 sequences from either both copies of der(8) or from the der(12), including a deletion at 8q12.1 at the LYN locus). This suggests localised chromothrypsis on chromosome 8 but this cannot be confirmed without sequencing.

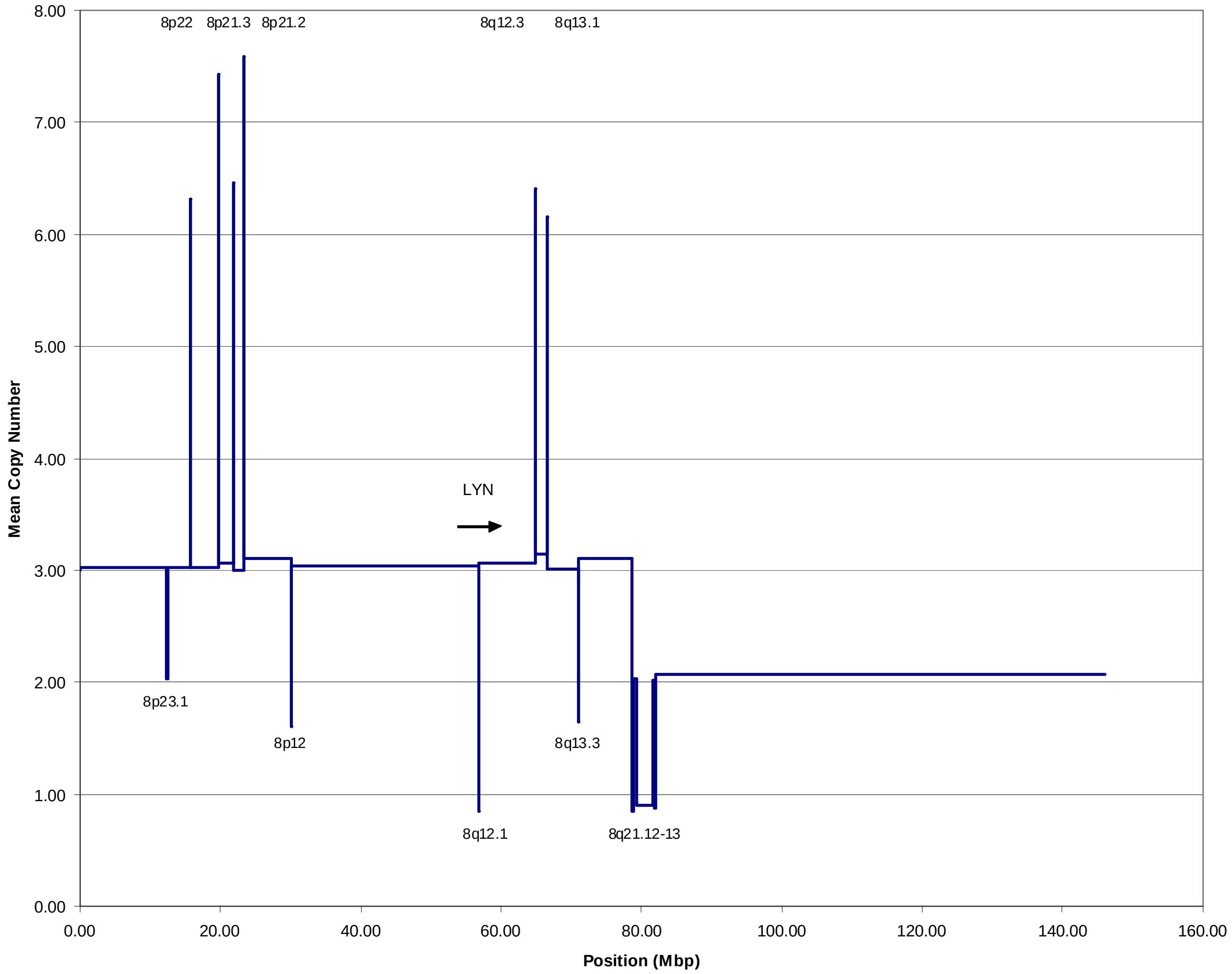

Reverse transcriptase PCR

Supplementary Method 1

Genomic DNA was extracted from bone marrow mononuclear cells, from diagnosis and remission, using the QIAamp® DNA Blood Maxi Kit (Qiagen). Total RNA was extracted from bone marrow mononuclear cells from the patient using the RNeasy Plus kit (Qiagen). RNA was tested for the *ETV6-Lyn* fusion by reverse-transcriptase PCR, using specific primers for the *ETV6* and *Lyn* genes (as described in Tanaka et al, 2009). The *ETV6-AML1*-positive acute lymphoblastic leukaemia cell line REH was used as a negative control. A 778bp band was detected in the patient sample, but not in the REH cell line. The PCR product was cloned into pCR2.1 using the TOPO TA cloning kit (Invitrogen) and subsequently sequenced using M13 forward and reverse primers.

Primers:
ETV6-F: ccctcccaccattgaactgt
LYN-R: gcgtagagcctcacgagctt

Figure 5.

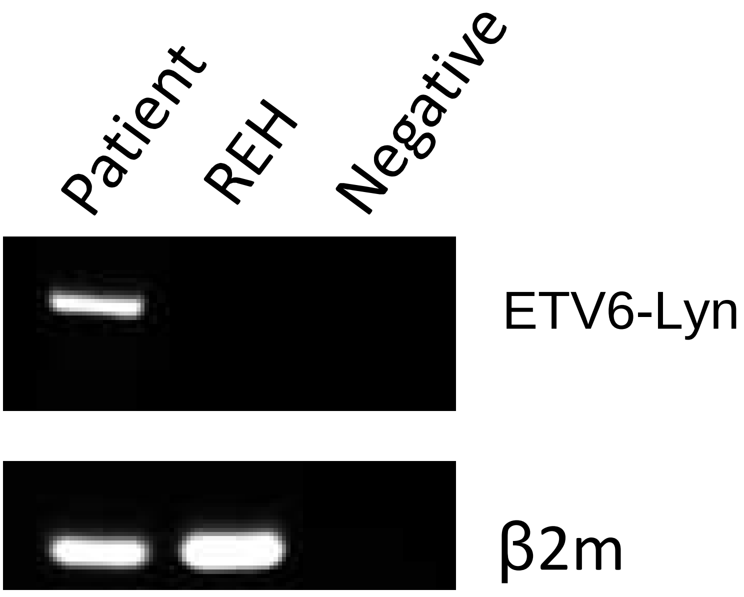

Figure 6. Capillary sequencing of RT-PCR product

ETV6-LYN PCR product

CCCTCCCACCATTGAACTGTTGCACCGCTCCAGGTCACCTATCACGACAAATCA
CCGGCCTTCTCCTGACCCCGAGCAGCGGCCCCTCCGGTCCCCCCTGGACAAC
ATGATCCGCCGCCTCTCCCCGGCTGAGAGAGCTCAGGGACCCAGGCCGCACC
AGGAGAACAACCACCAGGAGTCCTACCCTCTGTCAGTGTCTCCCATGGAGAAT
AATCACTGCCCAGCGTCCTCCGAGTCCCACCCGAAGCCATCCAGCCCCCGGCA
GGAGAGCACACGCGTGATCCAGCTGATGCCCAGCCCCATCATGCACCCTCTGA
TCCTGAACCCCCGGCACTCCGTGGATTTCAAACAGTCCAGGCTCTCCGAGGAC
GGGCTGCATAGGGAAGGGAAGCCCATCAACCTCTCTCATCGGGAAGACCTGG
CTTACATGAACCACATCATGGTCTCTGTCTCCCCGCCTGAAGAGCACGCCATGC
CCATTGGGAGAATAGCAGAGCAGGCAGATGGCTTGTGCAGAAGATTGGAGAAG
GCTTGTATTAGTCCCAAGCCACAGAAGCCATGGGATAAAGATGCCTGGGAGAT
CCCCCGGGAGTCCATCAAGTTGGTGAAAAGGCTTGGCGCTGGGCAGTTTGGG
GAAGTCTGGATGGGTTACTATAACAACAGTACCAAGGTGGCTGTGAAAACCCTG
AAGCCAGGAACTATGTCTGTGCAAGCCTTCCTGGAAGAAGCCAACCTCATGAA
GACCCTGCAGCATGACAAGCTCGTGAGGCTCTACGC

Key

Etv6

Lyn

Fusion occurs between *ETV6* exon 5 and *LYN* exon 8.

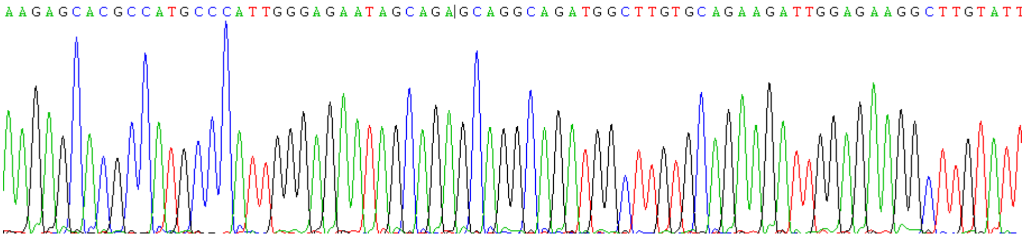

Figure 7. Suggested mechanism of formation of ETV6-LYN fusion

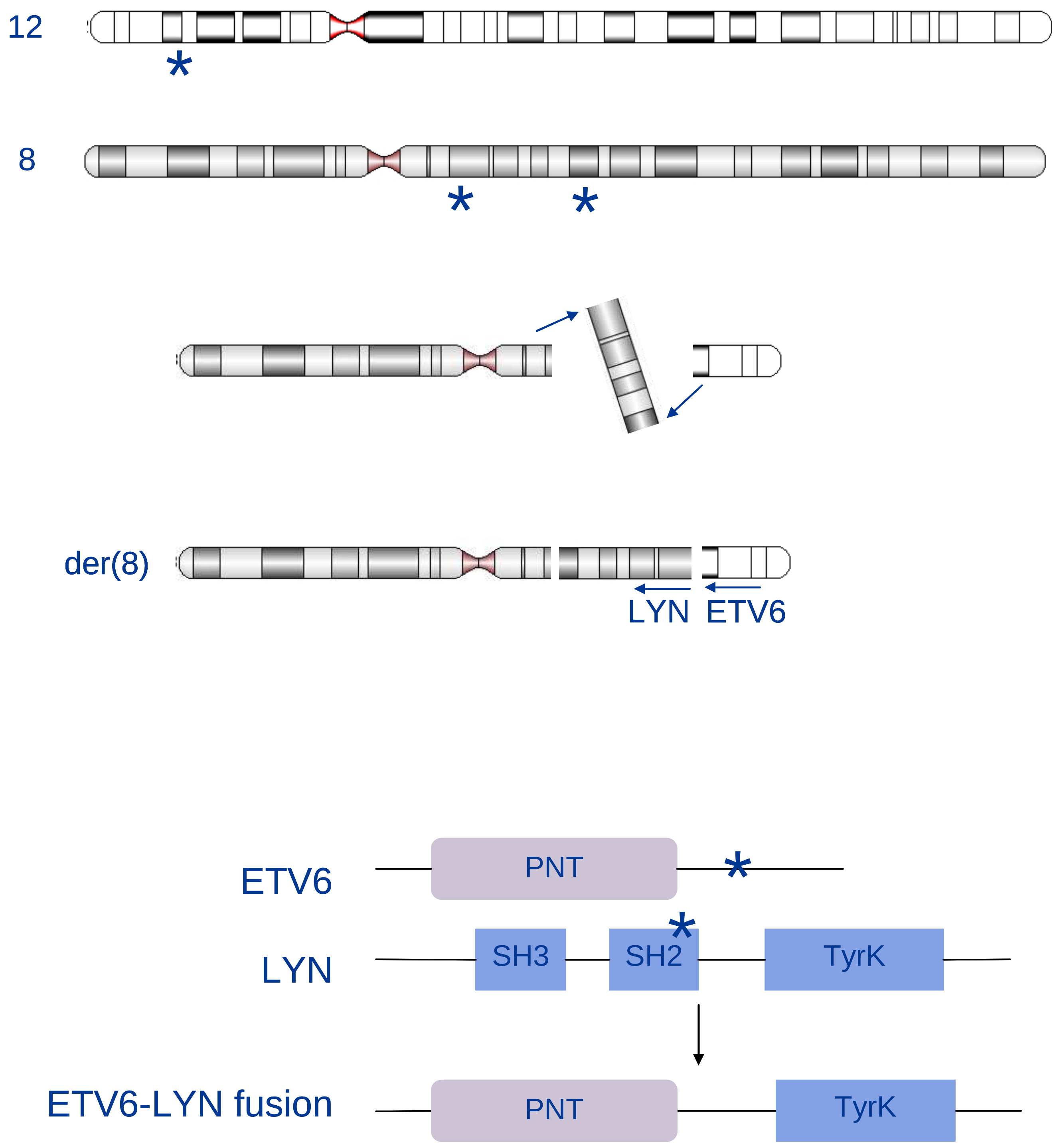

Table 1. Comparison of similar cases of ETV6-LYN / t(8;12) stem cell leukemia/lymphoma syndrome

Case	Age/ sex	Presentation	Presentation FBC	Marrow	Lymph nodes	Karyotype	Treatment	Survival
**Current**	46 male	Bilateral inguinal lymphadenopathy. Splenomegaly.	WCC 17.2 $x10^9$, Hb 94 g/l, Plts 184 $x10^9$. Neutrophils 9.1 $x10^9$, Eosinophils 4.0 $x10^9$. Metamyelocytes 1.0 $x10^9$, myelocytes 1.2 $x10^9$, promyelocytes 0.2 $x10^9$.	Aspirate; Myeloid hyperplasia with eosinophilia. Raised myelocytes and neutrophils, and eosinophils. No excess of blasts or basophils. No evidence of lymphoma. Fibrosis not reported. Trephine; myeloid hyperplasia with marked increase in eosinophils. 10% interstitial infiltrate of CD2+ CD3+ T-cells. TdT scantly +ve.	Precursor T-lymphoblastic lymphoma*<br><br>CD20-, CD79a +/-, CD2+, CD3+, CD5+, CD10- Bcl2 +/- Bcl6- TdT +ve High Ki67 proliferative fraction (80%)	der(8)inv(q12.1q21.1) t(8;12)(q12.1;p13), der(12)t(8;12)(q12.1;p 13)	DDVA chemo FLA-IDA chemo Intensive chemo following transformation to AML	~7 months Transformation to AML
**Tanaka et al 2010**	21 male	Abdominal mass, pain, fever. Splenomegaly.	WCC 25 $x10^9$ Hb 69 g/l Plts 24 $x10^9$ leucoerythroblastosis	Dry tap Raised reticulin. PMF	-	Ins(12;8)(p13;q11q21)	Chemotherapy Splenic irradiation Allo PBSCT from HLA identical brother x2	~11 months Blastic transformation
**Shvidel et al 2008**	47 male	Lymphadenopathy. Splenomegaly.	-	Myeloid hyperplasia with eosinophilia Raised reticulin No evidence of lymphoma	T-lymphoblastic lymphoma Tdt +ve, CD1a, CD2, CD5	t(8;12)(q11;p12)	Chemotherapy Methotrexate and cytarabine SCT following transformation to AML	~5 months Transformation to AML

Supplementary Method 2.

TKI Sensitivity studies

Method

Drug sensitivity was analysed using a modification of the Promega CellTox™ Green (Promega, Madison, Wisconsin) cytotoxicity assay. This assay utilises a cell impermeant dye that detects fragments of DNA released from damaged cells into the extracellular media. Due to the long term stability of the reaction, the assay can be monitored continually. Test and control cells were washed and resuspended at a density of $2x10^5$/ml in phenol red-free RPMI supplemented with 10% FCS prior to labeling with CellTox Green Dye. Cells were seeded in triplicate in wells of a 96 well plate and test compounds were added over a range of concentrations from 1nM – 10µM. Fluorescence was measured (Ex 485nm/Em 520nm) every 30 minutes for 72 hours using a BMG Labtech Fluorstar with climate control to maintain the cells at 37°C and 5% CO_2. The assay was validated by retrieving expected results with the myelogenous leukaemia cell line K562 and a TKI resistant daughter K562 cell line that has been cultured for long periods with low doses of TKI to generate a resistant phenotype (data not shown). In order to compare results across different plates and separate assays, the data was standardized to account for variation in dye uptake and the stability and relative capacity of different cell types. As an internal positive control, triplicate wells were treated with Promega cell lysis buffer at the start of the assay, to make the entire DNA available to the dye. This established fluorescent values so that data was expressed relative to maximum cell death. The data was then further normalized, by expressing it relative to the DMSO-only control (untreated) cells.

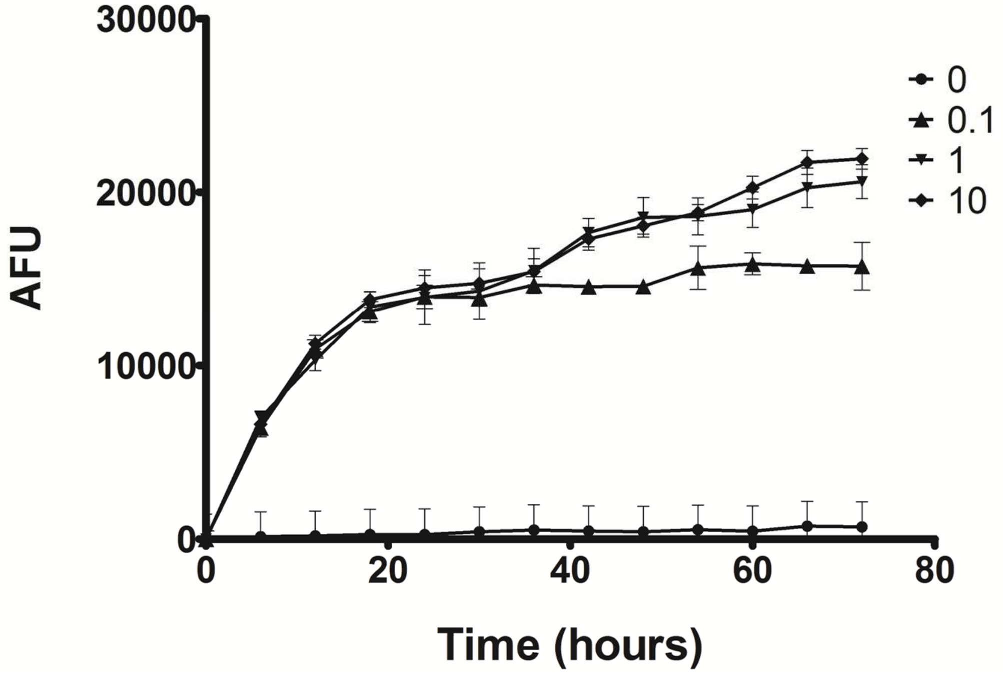

Figure 8. Sensitivity of cells from the diagnostic bone marrow with various concentrations of dasatinib (0.1, 1 and 10 μM) (showing only readings at 6 hour intervals for clarity of display). Data is shown un-normalised to demonstrate error bars of 95% confidence interval using Tukey's Multiple Comparison Test and so cannot be compared directly to the remission sample. The graph shows that dasatinib has a significant effect at all doses and that both low and high doses appear to have a similar effect on cell viability.

One-way ANOVAR and Bartlett's test for equal variances showed a significant difference between treatments (P value <0.0001). Tukey's Multiple Comparison Test revealed significant difference between all concentrations of dasatinib and untreated ($p<0.05$) but no significance between the effect of different concentrations ($p>0.05$).
